# Borderline personality disorder and moral responsibility

**DOI:** 10.1007/s11019-024-10243-6

**Published:** 2025-01-04

**Authors:** Agnès Baehni

**Affiliations:** Université de Genève, Genève, Switzerland

**Keywords:** Borderline personality disorder, Control condition on moral responsibility, Epistemic condition on moral responsibility, Aggressions in borderline personality disorders

## Abstract

This paper seeks to determine the extent to which individuals with borderline personality disorders can be held morally responsible for a particular subset of their actions: disproportionate anger, aggressions and displays of temper. The rationale for focusing on these aspects lies in their widespread acknowledgment in the literature and their plausible primary association with blame directed at BPD patients. BPD individuals are indeed typically perceived as “difficult patients” (Sulzer 2015:82; Bodner et al. 2011), significantly more so than schizophrenic or depressive patients (Markam 2003). The “responsibility question” for patients with BPD has already been raised (Martin 2010; Zachar and Potter 2009; Bray 2003), but this paper tackles it from a novel perspective. First, I narrow down the category of things for which the responsibility question is *specific* to individual with BPD. After that, I argue that some of the diagnosis criteria of BPD such as emotional instability or impulsivity might serve as excusing factors targeting the “control condition” on moral responsibility. Second, this paper also considers another widely accepted condition on moral responsibility: the epistemic condition. The view defended in the paper is that the answer to the responsibility question for individuals with BPD, concerning both the control condition and the epistemic condition, hinges on an understanding of their epistemic profile.

##  Introduction

This paper seeks to determine the extent to which individuals with borderline personality disorders can be held morally responsible for a particular subset of their actions: disproportionate anger, aggressions and displays of temper. The rationale for focusing on these aspects lies in their widespread acknowledgment in the literature^1^ and their plausible primary association with blame directed at BPD patients. BPD individuals are indeed typically perceived as “difficult patients” (Sulzer 2015: 82, Bodner et al. 2011), significantly more so than schizophrenic or depressive patients (Markham 2003).^2^

The “responsibility question” for patients with BPD has already been raised (Martin 2010; Zachar and Potter 2010; Bray 2003), but this paper tackles it from a novel perspective. First, I narrow down the category of things for which the responsibility question is *specific* to individual with BPD. While it is intuitive that most of us are morally responsible for acting aggressively, it is unclear whether the same holds of individuals with BPD. As I will explain, some of the diagnosis criteria of BPD such as emotional instability or impulsivity^3^ might serve as excusing factors targeting the “control condition” on moral responsibility. The control condition on moral responsibility claims that S is responsible for x only if S had control over the happening of x (Fischer & Ravizza 1998). This condition has, implicitly or explicitly, been at the center of the debate of whether people with certain personality disorders could be held responsible for their actions. When we ask whether psychopaths should be blamed, for instance, we often wonder whether they can help it.

Second, this paper also considers another widely accepted condition on moral responsibility: the epistemic condition. This condition posits that for S to be held responsible for x, S must possess a certain knowledge or understanding of what they are doing (Harman 2011; Mason 2015; Rosen 2003; Levy 2014).

The view defended in the paper is that the answer to the responsibility question for individuals with BPD, concerning both the control condition and the epistemic condition, hinges on an understanding of their epistemic profile. People with BPD exhibit various pathological cognitive patterns, for instance paranoid ideation and dichotomous thinking. As we shall see, dichotomous thinking manifests as the inclination to make extreme interpersonal evaluations (“This person is a monster!”) while paranoid ideation is the tendency to believe that one is being threatened by others and their malevolent intentions (“I am certain that the nurse is trying to hurt me!”). It thus becomes plausible that individuals with BPD do not meet the epistemic condition for moral responsibility (Kyratsous & Sanati 2016: 974), challenging the common assertion that they precisely "know what they are doing."

The discussion also extends to the degree of *control* individuals with BPD have over their beliefs, focusing on the epistemic dimension of the control condition on moral responsibility. The conclusion of the paper highlights the fact that while BPD patients are not necessarily devoid of *indirect* control over their beliefs and actions (Levy 2011, chap. 5, Meylan 2015) several conditions must obtain. Insofar as these conditions are only rarely met, BPD patients should not be held to the same degree of responsibility for their aggressions as neurotypical individuals. 

The plan of the paper is as follows: In §1, a definition of borderline personality disorders is given. In §2, the focus shifts to the emotional instability of individuals with BPD, specifically addressing anger and aggressivity. §3 delves into the potential impairment of empathy in individuals with BPD, §4 examines their pathological thinking patterns, exploring tendencies for dichotomous thinking and paranoid ideation. § 5 provides some background on the notion of moral responsibility. In §6, the discussion extends to the question of whether individuals with BPD can control their frequent aggressions as well as their pathological thinking patterns, addressing the control condition on moral responsibility. In §7 I shift focus to the epistemic condition on moral responsibility. Finally, I conclude in §8.

While this paper is, to my knowledge, the first attempt to provide a detailed philosophical analysis of the extent to which individuals with BPD meet the conditions for moral responsibility, it is also driven by practical concerns. Families and caregivers face significant challenges in navigating the tumultuous emotional lives of individuals with BPD, which can dramatically affect behavior and relationships (Giffin 2008). Often, they find themselves grappling with whether to attribute responsibility and blame to their loved ones with BPD or to exempt them due to their condition. Hopefully, the present discussion will shed some light on these issues.

## What is borderline personality disorder?

Borderline personality disorder is a complex mental disorder that seriously impacts the quality of life of those affected by it and that usually develops during early adulthood (Mishra, Rawekar and Sapkale 2023: 3). Its severity is underscored by a correlation with important emotional instability, impulsive and self-harming behaviors as well as with recurrent suicidal behavior (Stanley & Brodsky 2005). Consider for instance the fact that the mortality rate by suicide is extremely high (Mishra, Rawekar and Sapkale 2023: 1): 10% of individuals with BPD commit suicide, that is a rate 50 times higher than in the general population (Skodol 2005: 3). This disorder is also relatively prevalent with estimates placing its prevalence between 1 and 2% in the general population. In addition, approximately 10% of all psychiatric outpatients and 15%–20% of inpatients are estimated to have BPD (Skodol 2005: 3, Widiger & Weissman 1991; Swartz et al. 1990). Therefore, this is a relevant problem that includes a considerable part of patients’ population.

BPD comprises no less than 9 diagnostic criteria, five of which are necessary to establish a diagnosis (Mishra, Rawekar and Sapkale 2023: 4). The criteria are as follows:

1. Frantic efforts to avoid real or imagined abandonment.
2. A pattern of unstable and intense interpersonal relationships characterized by alternating between extremes of idealization and devaluation.
3. Identity disturbance: markedly and persistently unstable self-image or sense of self.
4. Impulsivity in at least two potentially self-damaging areas (e.g. spending, sex, substance abuse, reckless driving, binge eating).
5. Recurrent suicidal behavior, gestures or threats, or self-mutilating behavior.
6. Affective instability due to a marked reactivity of mood (e.g. intense episodic dysphoria, irritability, or anxiety usually lasting a few hours and rarely more than a few days).
7. Chronic feelings of emptiness.
8. Inappropriate, intense anger or difficulty controlling anger (e.g. frequent displays of temper, constant anger, recurrent physical fights).
9. Transient, stress-related paranoid ideation or severe dissociative symptoms.

Criterion 1 highlights the tendency of individuals with BPD to perceive an impending abandonment from those close and significant to them (Skodol 2005: 5, Palihawadana 2019, Matthies et al. 2018). Criterion 2 is about the fact that BPD patients often grapple with exceptionally unstable relationships, particularly with those closest to them, such as family members, roommates, or romantic partners (Scott Lori et al. 2017:1). BPD patients typically invest heavily in their close relationships and exhibit a tendency to swiftly idealize the person. Disappointment with this individual, even at minor levels within close relationships, can trigger a rapid shift to devaluing the other person, who is now perceived as not caring nearly enough (Skodol 2005: 6; Jeong et al. 2022). As we shall see, such dramatic shifts often occur in response to the perception of rejection or abandonment. Criterion 3 will be discussed in the last part of the paper: individuals with BPD have exceptionally unstable self-images which can lead to further interpersonal difficulties such as failing to accept blame and taking responsibility. Criteria 4, 5, 6 and 7 are relatively straightforward. Further insights into criterion 8 will be provided in §3, where the focus shifts to aggression in BPD patients. Paranoid ideation (criterion 9) entails the feeling of being threatened or persecuted. For example, a BPD patient may believe that nurses are conspiring against her.

These 9 diagnostic criteria conjure up a number of different concepts. There are (at least) three categories of pathological symptoms in BPD: symptoms related to *pathological actions*, symptoms tied to *pathological emotional regulation*, and symptoms associated with *pathological thinking patterns*. The following table sums up these observations:

**Table:** 

*Emotional* aspects of the diagnosis criteria	*Epistemic* aspects of the diagnosis criteria	*Action-tendency* aspects of the diagnosis criteria
Chronic feeling of emptiness (criterion 7) Emotional instability (criterion 6) Intense anger (criterion 8)	Imagined abandonment (criterion 1) Extreme idealization and devaluation (criterion 2) Paranoid ideation (criterion 9)	Frantic efforts to avoid real or imagined abandonment (criterion 1) Impulsive behaviors (criterion 4) Frequent display of temper, physical fights (criterion 8) Recurrent suicidal behaviors (criterion 5)

Concerning the epistemic dimension of the disorder Veen & Arntz 2000 note that “Three basic schemata are assumed to play a central role in BPD. These can be summarized by the following assumptions: ‘‘The world is (i.e. others are) dangerous and malevolent’’, ‘‘I am powerless and vulnerable’’, and ‘‘I am inherently bad and unacceptable’’ (Beck et al. 1990, quoted in Veen & Arntz 2000: 24). A schema or schemata is a type of belief structure around which the individual understanding of the world is organized (Sieswerda et al. 2013:36–37). I will argue that it is precisely the influence of these schemata on individuals with BPD that leads to difficulties in reaching verdicts of moral responsibility.

Adding to the complexity of this disorder is its classification within diagnostic cluster B. In recent literature, this category is frequently associated with moral rather than solely medical impairments (Zachar & Potter 2010, Horne 2013). Personality disorders are typically divided into three clusters. Cluster A encompasses *odd/eccentric disorders*, such as paranoid personality disorder or schizoid personality disorder. Cluster B includes disorders characterized as *dramatic/erratic*, such as borderline personality disorder, antisocial personality disorder, and narcissistic personality disorder. Lastly, cluster C comprises disorders classified as *anxious/inhibited*, such as dependent personality disorder or avoidant personality disorder.

An intriguing yet perplexing aspect of this classification is that the disorders in clusters A and C can be defined without referring to any moral terms. By contrast, disorders in cluster B incorporate references to moral terms in their definitions. For instance, borderline personality disorder includes a tendency towards *disproportionate anger.* Antisocial personality disorder is defined by the propensity for *violent behavior* towards others *without displaying guilt* (Black 2015,) and individuals with narcissistic personality disorder exhibit a tendency to *exploit* those who admire them, among other traits (Yakeley 2018).

As a result, some philosophers have suggested the controversial idea that members of diagnostic cluster B are not medical conditions but rather moral conditions, arguing that the treatment they require is not medical but moral (Charland 2004). This is a controversial view, but it does not mean (at least as I read it), that individuals with BPD can never be exempted from moral responsibility, unlike “real” medical patients. Rather, this view emphasizes that the distinction between the moral and the medical is not always sharp, especially when it comes to cluster B disorders and is silent on whether they can be morally responsible.

Zachar and Potter note that “the diagnosis cannot rid itself of saturated moral value, but (…) it may have indispensable nonmoral features that allow us to classify it as a mental disorder” (2010: 102). One such non-moral feature is the fact that BPD individual suffer intensely from their conditions, since they constantly fear being abandoned by those they love. Their disorder is also quite clearly rooted in a conjunction of genetic and environmental factors. Thus, the fact that the diagnosis of BPD refers to moral notions does not prevent its classification as a medical condition and leaves the responsibility question open.

As these admittedly brief remarks indicate, BPD is located at the intersection of fundamental and interconnected moral questions. To allow clinicians, patients and families to approach challenging situations with the right conceptual tools, we should thus strive to clarify these issues. As a first step in this direction, the next section discusses the prevalence of aggression and disproportionate anger in BPD.

## Anger management in BPD

Diagnostic criterion 6 for borderline personality disorder refers to borderline patients' difficulty in controlling their anger and aggression levels. These difficulties can manifest in a number of ways, some self-directed and some directed outwards, towards objects or people (Cackowski et al. 2017: 2). These intense emotional episodes are often caused by and directed at others whose behavior individuals with BPD interpret as “being neglectful, withholding, uncaring, and abandoning” (Skodol 2005: 6). In his seminal work on the treatment of BPD patients, Kernberg showed that aggressive behavior and affect in BPD individuals finds deeper roots in severe trauma during early childhood, such as physical or sexual abuse (Kernberg 1994). In their meta-analysis of 97 studies, Porter et al. (2020) also found that 95% of individuals with BPD reported difficult childhoods.

As indicated, individuals with BPD have a propensity toward anger and aggression. These frequent displays of anger and aggression can be particularly difficult for family members and caregivers, hindering both the patient's healing process in therapy and his or her social integration (Scott et al. 2017:1). This symptom thus does not come without a cost for the individual as it prevents them both from receiving quality care and from forming fulfilling social bonds (Neukel et al. 2022: 2).

It is somewhat difficult to define aggression, but I shall use as a starting point the definition suggested by Scott et al. (2017: 1): “Aggression, defined as behavior intended to harm another person, emotionally, socially, or physically”. This definition fits our pre-theorical understanding of aggression with the exception that we might want to count as aggressions some behaviors that are not properly *intended* to harm, maybe because they are very impulsive, but that still have such an effect.

BPD patients are often referred to as difficult patients, so much so that they represent a real challenge for therapists. To those around them, these patients can also project an image of being violent, aggressive and mean. As an illustration, consider the following exchange from Nancy Potter’s discussion of manipulation in BPD, where Ms A is a patient with BPD (2006: 140):

Ms A: You could be dying before you got any help around here! My arm is killing me! This place is crazy!

Therapist: Ms. A, I would like to introduce myself. I am Dr. Wheelis.

Ms. A: Oh, no kidding! I didn’t expect you. You’re a resident? Interesting. You must be either very good or very crazy to have taken me on.

Therapist: I can’t tell if that’s an invitation, a warning, or both (patient smiled at the comment), but we have an appointment tomorrow. For now, perhaps I should take a look at your arm.

Ms. A: No, it’s okay, just a little bang.

This situation, although relatively mild, illustrates how patient with BPD can display anger and aggressivity through sarcasm towards a medical professional. But, unfortunately, BPD patient’s difficulty to regulate their anger can also manifest in more dramatic situation.

In their study about anger and aggressivity in BPD, involving 117 adult women between 18 and 24 years old with recent history of aggressivity, Scott et al. found that 66.7% of the participant reported “Aggressive urges, threats, or behaviors” (2017: 8) at least one time during the time of assessment (three weeks) (2017:8). The study showed that aggressive behaviors were almost always directed at others “within close relationships (romantic partner or ex-partner: 48%; family member, friend, or roommate: 39%)” (2017:8). These findings are consistent with other diagnostic criteria for borderline personality disorder, notably criterion 2, which refers to the fact that patients with BPD have extremely tumultuous close interpersonal relationships. Typically, BPD individuals react the most aggressively towards those from whom they expect care and attention, which, tragically, prevents them for entering the satisfying and securing close interpersonal relationships they need. BPD individuals fear that they will be abandoned by their loved ones from whom they expect to be constantly reassured. When these demands are not met, BPD individuals typically experience strong emotional responses which in turn lead them to react with anger and aggressivity (Scott et al. 2017: 2). On the long run, one can see how these violent reactions can make their worst fear come true: that the people they care about will distance themselves.

A final point to address is the connection between BPD and criminal behavior. In their review of the literature on BPD and criminality Sansone & Sansone observe that a significant proportion of inmates suffer from BPD (2009: 16). Citing Jordan et al. (2016) and their study involving female inmates, they observe that, while individuals with BDP represent approximately 2% of the population, they represent as much as 28% of the female inmate population. Another study by Riesco et al. 1998 (also quoted in Sansone Randy and Sansone 2009) about male inmates found that 41% of the inmates have BPD. The overwhelming majority of studies cited found a strong correlation between criminality and BPD. Sansone & Sansone (2009: 18) conclusions are that most studies land support to an important asymmetry in BPD rates between prison population and regular communities with BPD individual sometimes forming as much as 50 percent of prison inmates. In studies comparing men and women’s rates of BPD in prison population, they found that significantly more female than male inmates could be diagnosed with BPD, an asymmetry that is still to be explained (2009: 18).^4^ We can thus see a clear connection between a diagnosis of BPD and higher levels of anger and aggression. It is also worth noting that these episodes of anger often stem from feeling rejected by loved ones. In the next section, I turn to one possible way of explaining high levels of aggression in individuals with BPD: a potential impairment in empathy.

## Lack of empathy in BPD

Here, I briefly discuss a hypothesis that could explain the violent tendencies of patients with BPD: an empathy impairment. Drawing an analogy with antisocial personality disorder, which also falls within diagnostic cluster B, we might attempt to explain the violent tendencies of both types of personality disorders through a similar impairment in empathy. It is widely acknowledged that the violent acts of patients with antisocial personality disorder can be attributed to their inability to feel empathy (Shoemaker 2015: 146; Gao and Adrian 2010). This explanation could potentially be extended to patients with BPD.

There are, broadly speaking, two meanings to the word empathy in the psychological literature. The first is “cognitive empathy”, that is, the ability to adopt someone else’s perspective (Shoemaker 2015: 146). The other one is “affective empathy” (Shoemaker 2015: 146). In a nutshell, affective empathy is the ability “to feel what others feel” (Shoemaker 2015: 146﻿). It is this second form of empathy that is at stake in assessing the moral responsibility both of antisocial personality disorders and BPD. Do BPD patients, like antisocial personality disorder, suffer from an impairment in affective empathy?

The short answer is “no”. Recent empirical findings show that individuals with BPD have a higher capacity for empathy than neurotypical individuals. This is paradoxical, especially in light of the fact that they typically encounter difficulties in maintaining stable and fulfilling interpersonal relationships (Dinsdale et al. 2013: 172). This increased empathy might be explained by the necessity for the borderline patient to maintain a stable image of those whom they perceive as their caregiver. In order to maintain such a stable image, the BPD individual develops enhanced sensitivity to the subtle, subconscious cues indicating the mental states of the person (Dinsdale et al. 2013: 187). This could explain, at least partly, BPD patients’ propension to aggressivity as they will often perceive hostility that is either absent or so mild that others would not notice it (Ripoll et al. 2013: 6). Typical examples include individuals with BPD perceiving a potential abandonment or hostility if their loved ones don’t answer their texts fast enough, if they disagree with them or cancel a plan.

In light of the findings discussed in this section, it seems implausible that BPD individuals’ tendency for anger and aggression could be explained by an empathy deficit, rather the opposite. In the next section, I discuss two pathological reasoning patterns typical of individuals with BPD that could provide us with a fuller picture of the difficulties encountered by individuals with BPD in relating to others: dichotomous thinking and paranoid ideation. As we shall see, they might be especially helpful to explain BPD individuals’ proclivity towards violence and aggressivity.

## Dichotomous thinking & paranoid ideation

### Dichotomous and negativistic thinking

As Matt King and Joshua May rightly remark: “the diversity of the ways in which the symptoms of mental disorders affect action makes them an extremely heterogenous class, such that there is no supported general inference from having a disorder to any claim about moral responsibility” (2018:20). Before drawing any conclusion, it is thus important to get clear on the link between symptoms and action. Let us begin with the symptom usually referred to as “dichotomous thinking”.

Dichotomous thinking is the inclination to “evaluate experiences in terms of mutually exclusive categories rather than to see experiences as falling along continua” (Veen & Arntz 2000: 23). It is consistent with criterion 2, according to which individuals with BPD are especially prone to alternating between extremes of idealization and devaluation. Dichotomous thinking is characteristic of BPD individuals (Sieswerda et al. 2013) and, like other of their symptoms, these schemata stems from traumatic experiences during childhood, often involving care givers (Veen & Arntz 2000: 24).^5^

Another resembling schemata that is helpful in characterizing borderline personality disorder is negativistic thinking (Sieswerda et al. 2013: 37). Negativistic thinking is the tendency to make negative interpersonal evaluations. BPD patients tend to evaluate others more negatively than control groups, especially when presented with schemata related stimuli: “Finding negativistic thinking in BPD is in line with clinical observations of strong tendencies towards victimization and suspiciousness in this patient group (e.g. Kroll, 1988)” (2013: 47). These remarks are in line with studies that found that BPD patient typically evaluate others more negatively than neurotypical people when presented with different stimuli like movie characters, facial expressions, ink blots etc. (2013: 47).^6^ Importantly, negativistic evaluations in BPD patients are worse when it comes to schemata-related characters, for instance a parent or a romantic partner. (2013: 48).

It thus seems that BPD patients exhibit two tendencies in their interpersonal evaluations: first, they tend to make extreme evaluations and, second, they tend to typically evaluate those around them more negatively than control groups. Taken together, these two inclinations conjure up a picture on which BPD patients tend to make extremely negative evaluations of those they perceive as being ill-intentioned.

Before discussing the relation of dichotomous and negativistic thinking to anger and aggression I say a few words about paranoid ideation.

### Paranoid ideation

Paranoid ideation refers to the persistent belief or suspicion that others intend harm or malevolence towards oneself, despite lacking sufficient evidence to support such beliefs (Beck, Aaron.T., Freeman, Arthur., & Associates 1990). Paranoid ideation is often accompanied by a difficulty to trust others, a sense of vulnerability (cf. the schemata discussed in the introduction) and a readiness to defend oneself through aggressive behaviors (Gilbert et al. 2005: 125). Coid et al. for instance (2016) observed a strong connection between paranoid ideation and violence that couldn’t be explained by other psychiatric comorbidities.

Paranoid ideations often lead individuals with BPD to interpret neutral or benign interactions as threatening or hostile. It is interesting to note that the perception of mistreatment and neglect has been observed to be comparatively as high in BPD patient than in individuals with other personality disorders such as schizophrenia (Kingdon et al. 2010: 402). The underlying fear of abandonment, another core feature of BPD, may contribute to the development of paranoid thoughts, as individuals with this disorder may perceive any rejection or criticism as confirmation of their fears.

### Dichotomous thinking, Paranoid ideation and Aggressivity

Since individuals with BPD believe that others are malevolent (paranoid ideation) and, generally, tend to make more extreme and negative evaluations than neurotypical people (dichotomous or negativistic thinking), it does not seem far-fetched to link their readiness to display aggressive behaviors to these thinking patterns. Butler further observes that (2002: 1238): “Several of the beliefs associated with BPD patients appear to be not only dysfunctional, but contradictory as well. This internal dissonance may further contribute to the maladaptive behavior and distressed affective state exhibited by many BPD patients.” For instance, let us imagine that a BPD patient holds the belief that those close to them are trying to hurt them and are not to be trusted, but that, at the same time, he feels helpless and fears abandonment. It is not difficult to imagine how holding such contradictory and inherently painful beliefs can lead to violent emotions.

Having provided some empirical background on the symptoms and causes of BPD, we are now in a good position to consider whether BPD individuals should be held responsible for their frequent aggressions. In the next section, I begin by providing some theoretical background on the notion of moral responsibility. After that, I move on to discussing the control condition on moral responsibility in Sect. 6” and the epistemic condition in Sect. 7.

## Moral responsibility: some theoretical background

A common approach to understanding moral responsibility centers on the concept of basic desert (Menges 2023) and defines it as "the degree of control over one's actions necessary for those actions to truly deserve blame or praise" (Caruso 2021). Derk Pereboom explains that for an agent to be morally responsible in the basic desert sense for an action: 

“(…) is for the action to be hers in such a way that she would deserve to be blamed if she understood that it was morally wrong, and she would deserve to be praised if she understood that it was morally exemplary. The desert at issue here is basic in the sense that the agent would deserve to be blamed or praised just because she has performed the action, given an understanding of its moral status, and not, for example, by virtue of consequentialist or contractualist considerations (Pereboom 2014, 2).^7^

In the literature, this form of responsibility is often referred to as "responsibility as accountability" (Watson 2004). Holding someone responsible in the accountability sense has practical implications: they can be blamed, punished and subjected to attitudes like resentment and indignation. Thus, when we ask whether individuals with BPD should be held morally responsible, we are asking whether they merit to be blamed or punished.

However, it is important to note that moral responsibility can also be conceptualized differently. A well-known alternative is to understand moral responsibility as attributability, the key issue being then whether actions can be rightfully *attributed* to the subject. Attributability, for Watson, is the “aretaic face of responsibility”. When is an action *attributable *to someone? On most views, an action is attributable to someone when it reveals something about who they are, such as their commitments or values. For David Shoemaker for instance, an attitude can be attributed to someone if it “expresses (…) the agent’s cares, commitments, or care-commitments clusters (2015: 59). On the prominent quality of will view (Arpaly 2003), one can be held morally responsible for an action if it expresses a lack of concern for those it affects. One difference between responsibility as accountability and responsibility as attributability is that the former is concerned with the appropriateness of reactive attitudes such as blame, while attributability centers on attribution of virtues and vices. Since this paper aims to clarify how people should adjust their relationships with individuals who have BPD, for instance by renouncing blaming them, our focus will be on responsibility as accountability.

Different theories offer various answers to the question of what it takes to be an appropriate target for accountability practices. I will adopt a strategy similar to that of King and May in their discussion of mental illness and responsibility, where they “highlight a few features of agency that different theories of responsibility have emphasized, exploring the ways in which different disorders will be more or less threatening to those features” (2018:15). While King and May focus on choice, control, and coherence, I will concentrate on the control and epistemic conditions of moral responsibility. As is standard in the literature, I will consider these conditions to be necessary for the attribution of moral responsibility (Rudy-Hiller 2022; Montminy 2021: 168).

Up to this point, I have been speaking in a way that suggests that our practice of holding others responsible, at least neurotypical individuals, is justified. In this paper, I will not challenge this traditional approach to moral responsibility. Note however that recent findings in neuroscience and philosophy challenge this picture. Prominent philosophers have indeed advocated for skepticism about moral responsibility (Levy 2011, 2014; Pereboom 2014, 2022; Caruso 2021; Caruso & Pereboom 2022, Sommers 2009). Their reasons are varied, stemming from concerns about free will and determinism (Levy 2011 ), the neuroscience of decision-making processes (Nisbett and Wilson 1997; Kahneman 2011), and luck (Levy 2011). However, these skeptics do not necessarily reject the practice of holding others responsible—such as through blame—entirely; they merely deny that individuals can truly deserve it based on any backward-looking considerations (Zawadzki 2022). According to these views, our responsibility practices can still be justified by forward-looking considerations, such as the development of moral agency (Vargas 2013) and moral formation (Pereboom 2022).

While the forward-looking approach to moral responsibility is an exciting and promising field, I will nevertheless stick to the traditional view, mostly due to its intuitiveness. The argument I will present in the next two sections is thus conditional: if it is ever possible for anyone to be truly deserving of blame and praise, then the epistemic and control conditions are the things that needs to be fulfilled. The goal then, is to see if individuals with BPD can fulfill these conditions.

## Do individuals with BPD fulfill the *Control Condition* on Moral responsibility?

The idea behind the control condition is that one must be appropriately connected to one’s actions. The most popular approach takes this appropriate connection to be a matter of whether the agent acted for their own reasons (Wolf 1990; Nelkin 2011; Fischer and Ravizza 1998). On Fischer and Ravizza’s view, for instance, moral responsibility for an action does not presuppose that one could do otherwise; it is enough that one is responsive to reasons.

Now, there are two sides to reason-responsiveness. On the one hand, reason-responsiveness has a receptivity or recognitional component that requires the agent’s ability to form beliefs based on evidence. On the other hand, reason-responsiveness has a reactivity or motivational component that requires the agent to acquire the relevant motivational states, such as desires, emotions and volitions. As Pereboom and McKenna (2016: 219) explain: “Receptivity is the capacity to recognize and evaluate a spectrum of reasons for action, while reactivity is the capacity to act in accord with such recognition and evaluation of reasons”. This means that a responsible agent must be able to form beliefs based on his reasons and to be motivated to act according to such reasons.

As it is difficult to think about responsibility in the abstract, in this section and the next, I will anchor my discussion in the following case:

**Inheritance:** Mario is a young man diagnosed with borderline personality disorder (BPD). His condition is rooted in his early life experiences, particularly his abandonment by his biological parents when he was two years old. Following this trauma, Mario was adopted by a caring and loving family, who later had two additional children. As Mario grew older, his disorder manifested in frequent outbursts of aggression towards his siblings and adoptive parents, whom he accuses of "not loving him as much as his other siblings." He often blocks them on social media, shouts at them in public, and has even slapped his mother on several occasions. Additionally, Mario has developed paranoid fears, accusing his siblings and parents of attempting to "rob him of his money" and of "conspiring against him". Despite these beliefs, none of his accusations are true; his siblings and parents love him dearly and are deeply saddened by his tendency to perceive their actions as malevolent. As of today, Mario does not receive proper therapeutic help.

Does Mario fulfill the control condition on moral responsibility for slapping his mother and blocking his family on social media? Does he possess the receptivity and reactivity components of reasons responsiveness?

Let us start with the reactive component. One way to argue that individuals with BPD partly lack reactive or motivational control is by noting that, according to diagnosis criterion 4, they are very impulsive. In addition, we also noted that these individuals are prompt to experiencing disproportionate level of anger (criterion 8). This supports the claim that individuals with BPD may lack some motivational control. When in the grip of anger, they usually swiftly respond with aggression to what they perceive as harmful behavior, for instance by blocking their family on social media, ripping pictures apart, deciding to move out of the house, or break up, even when their loved ones do everything in their power to calm the situation. They might know that they shouldn’t scream in public or slap people, but they will do it nonetheless for the reasons we just mentioned such as their impulsivity. It is nevertheless important to note that saying that Mario partly lacks motivational control for his aggressions does not mean that Mario does not satisfy this condition concerning many of his other everyday actions.

So, individuals such as Mario lack motivational control on their aggressions insofar as these actions are not normally responsive to reasons. This is relatively uncontroversial: we usually regard people who lack motivational control due to their emotional immaturity and impulsivity, such as adolescents, at least somewhat responsible to a lesser degree.

However, the way in which BPD individuals fail to fulfill the control conditions runs deeper than the purely motivational level. Consider the receptivity or recognitional component (Pereboom & McKenna 2016: 219). As Neil Levy remarks, there is an important epistemic dimension to the control condition, having to do with the control we have over our belief acquisitions (Levy 2011: 112). The problem with BPD individuals is thus not only that they have difficulties in controlling their reactions, but also that they face difficulties in controlling their beliefs. Following Levy, if one’s beliefs are reasons-responsive and if one’s reasons for action are one’s beliefs, one’s actions are reasons-responsive (Levy 2011: 111). We will deny that individuals with BPD have such a capacity.

In earlier sections, we identified two cognitive patterns prevalent in BPD patients, dichotomous thinking and paranoid ideation, which could account for their inclination toward aggression. Some core beliefs of individual with BPD, such that others are generally malevolent and that their loved ones are on the verge of abandoning them are not responsive to reasons. This is why we have called them “basic schemata” in Sect. 1 . They are exceptionally hard to revise in the face of countervailing evidence. As an illustration, let us imagine that Mario’s parent would tell him “Of course we are not going to take your money away, we love you!”. Individuals with BPD are likely to respond with even more suspicion, fearing that this is only a strategy to prevent them from getting their share. So, if these cognitive patterns are inherent to the BPD diagnosis, not responsive to reasons and plausibly explain their conduct, does this mean that we should not hold them accountable for their actions?

One tempting stance is to exempt them entirely, attributing these thinking patterns solely to diagnostic criteria rooted, as we have seen, in childhood trauma and genetic causes. However, while Mario lacks direct control over his belief acquisition, he might still be able to exert *indirect* control over her beliefs (Levy 2011: 130), in a way that could ground moral responsibility (Meylan 2015). Mario lacks direct control over the belief that his family is malevolent and is trying to harm him, but he might have indirect control over it. For example, he might work on his underlying fear of abandonment with a therapist and, over time, come to revise his belief. Now imagine that Mario was offered professional help and that he decided to stop going because it was too time consuming and didn’t like that the therapist wasn’t always agreeing with him. In this case, Mario’s direct responsibility for believing his parents are malevolent remains limited due to his limited reason-responsiveness. However, he would still be indirectly blameworthy because he is directly blameworthy for refusing to attend therapy when it was available.

Levy defines indirect control as “control in virtue of having control over something else” (2011: 130) adding further that “So far as responsibility is concerned, there seems little reason to treat direct and indirect modifications in our doxastic states differently: full-blooded moral responsibility could as easily be the product of indirect as direct modification (…) (2011: 130)”. Indirect control could be relevant to revising pre-existing beliefs such as “people are generally malevolent” as well as to the formation of new ones such as “right now, my parents are being unfair towards me and love my siblings more”.

Under what circumstance could BPD exert indirect control over their pathological cognitive patterns? Addressing this question requires considering the broader context of their condition. It is plausible that not all BPD patients are equipped to exert indirect control over their beliefs, especially if they are unaware of their condition or lack access to adequate professional help. For those receiving appropriate therapeutic assistance, the responsibility question becomes pertinent. In such cases, it falls upon their therapist to elucidate the various facets of their condition and its underlying causes. Ideally, an individual would then be empowered to mitigate the effects of their cognitive patterns, though it is unlikely that they would disappear entirely. Understanding the cognitive mechanisms at play could enable them to temper some of their cognitive behaviors, for instance, refraining from interpreting every setback as an abandonment, or tempering extreme evaluations of those close to them. This is, in fact, the goal of the therapeutical process for BPD patients and in many cases, it has showed promising results. It now becomes increasingly clear that contrary to some common misconception, BPD patients are not beyond therapeutic help. Studies such as Stone’s (2017), focusing on the long-term follow-up of BPD patients (25–50 years) notably show that over two-thirds reach clinical remission. Dialectical-behavioral therapies for instance have been shown to be very efficient in mitigating most manifestation of the disorder, especially when combined with appropriate medication (Mishra, Raweka and Sapkale 2023; Choi-Kan, Finch, Masland et al. 2017). Such therapies include revising some of their core beliefs as well as learning to manage their emotional reaction and their aggression levels.

In conclusion, the intermediate answer to our initial question—whether individuals with BPD can be held accountable for their displays of aggression when it comes to the control condition—is nuanced. They can be held accountable to the extent that they are equipped with the necessary tools to indirectly revise their beliefs as well as to learn to manage their emotional reaction and aggressions. I have explained how dialectical-behavioral therapy can target the basic schemata discussed in Sect. 1.. It will also be useful to note that it can be very efficient in managing individuals with BPD’s reaction since it is focused on behavior, thus increasing their motivational control. For instance, they can learn how to avoid such and such situations, learn to take some distance from their own emotions and so on.

However, given that this scenario represents an ideal, and potentially rare, circumstance, it is plausible that BPD patients often bear diminished responsibility for their aggressive behaviors because they often fail to fulfill (even indirectly) the control condition on moral responsibility both on the recognitional and motivational aspect. I now move on to the epistemic condition on moral responsibility.

## Do individuals with BPD fulfill the *Epistemic Condition* on moral responsibility?

The epistemic condition on moral responsibility tells us that for S to be responsible for x, S must have some knowledge or understanding of what S is doing. There are two main ways in which one can lack knowledge or understanding: factual and moral.

Consider a paradigmatic case of *factual* ignorance: if S believes that she is pouring sugar into her mother's tea but is actually pouring cyanide because someone replaced the sugar with cyanide, she cannot be held morally responsible for her mother's death, as she was justified in believing it was sugar (Harman 2011: 448). In contrast, consider a paradigmatic case of *moral* ignorance: a father who believes it is permissible to save money for his son’s college tuition but not for his daughter’s. While it is clear that the person in the first scenario is excused, the situation with the father is more complex. On the one hand, he appears blameworthy for his sexist treatment of his daughter. On the other hand, his ignorance may not be entirely culpable, given that he was shaped by the sexist norms of his time. While it is widely accepted that factual ignorance can exculpate, extending this verdict to moral ignorance is thus more contentious (Harman 2011: 450). Before we address this question, we must first ask whether individuals with BPD are ignorant in one of the two ways.

At first glance, individuals with BPD possess factual knowledge; for example, they know they are shouting or hitting someone. Thus, one might argue that individuals with BPD cannot be excused on the grounds of factual ignorance. However, when we examine Mario’s case more closely, it becomes clear that while he may have some factual awareness of his actions, he remains ignorant of key elements of the situation: he does not know that his family has good intentions and is not trying to hurt him. Similar to a person unknowingly pouring cyanide into tea, Mario is unaware that his actions—his violent behavior—are directed toward people who are, in fact, benevolent. In this way, Mario lacks factual knowledge and, therefore, can be excused. This reasoning can be extended to many cases involving individuals with BPD, where paranoid ideations and negativistic thinking result in a form of factual ignorance that can serve as grounds for excuse. These symptoms of their condition render them particularly at risk to have inaccurate factual beliefs about their loved ones and caregivers. The story could end here if all cases of aggressions in BPD were due to factual errors.

However, there are also numerous instances where individuals with BPD do not believe their caregivers or family members are malevolent but still act aggressively toward them due to impulsivity. In such cases, they often fail to recognize their actions as morally wrong. Typically, they might say things like, “I have a right to express my feelings even if it hurts others,” or “People should accept me as I am, unconditionally,” or dismiss the harm with statements like, “It’s not a big deal.” They may also shift the blame to others when confronted. As Sieswerda et al. (2013: 47) explain, individuals with BPD exhibit a strong tendency toward self-victimization, making it harder for them to take responsibility for their actions. This frequent failure to take responsibility is well documented^8^ and can be explained by the unstable self-image of individuals with BPD (Leichsenring et al. 2024). Admitting that they are wrong is nearly impossible for them insofar as doing so threatens their already fragile sense of self (criterion 2). In such cases, it seems clear that the kind of ignorance that is at stake is moral rather than factual ignorance: it is about what is permissible and what is not. So, the question essentially becomes under what circumstance one is blameworthy for one’s moral ignorance. This question has been much discussed. In what follows I offer a brief summary of the existing views.

There exist different answers to the question of whether and when moral ignorance exculpates. They can be, roughly, classified in three categories. The first category comprises views such as Rosen’s (2003) and Zimmerman’s (1997) on which an agent is only culpable for acts done out of ignorance if he is culpable of some action that “directly caused the agent’s current ignorance” (Mason 2015: 3039). On the proposed view, one can be *derivately* responsible for acts done out of ignorance if one is *directly* responsible for one’s ignorance. In turn, one’s ignorance is culpable only if it stems from a case of clear-eyed akrasia. As Fitzpatrick notes “we don’t get culpability until we arrive at a relevant episode of clear-eyed akrasia—either in the present action or in antecedents that contributed to the ignorance involved in the present actions” (2008: 593).

In my opinion, these views are not demanding enough. As others (Fitzpatrick 2017: 31) have noted, it lets too many people off the hook as we only rarely do wrong while thinking it is wrong. Rapist, for instance, often seem to think that their victim “deserved it” or that they were “asking for it”. On this view, people can be accused of being culpably morally ignorant only when they voluntarily cause their own ignorance. One example could be that of someone who, knowing that their victim will probably confront them about what they did, decides to block her on social media to avoid having to face difficult truths.

However, it is evident that we are often culpably morally ignorant even when we cannot be accused of having voluntarily caused our ignorance. For instance, many people think that it is morally permissible to drown kittens, a moral belief which seems to me to be culpable, even if they didn’t voluntary caused their own ignorance.

The second category of views admits that one can be morally responsible for an action while being ignorant of its moral significance as long as this action reveals something bad about one, such as, for instance, one’s insufficient moral concern for others. According to these views, blameworthiness presupposes a bad quality of will. Elizabeth Harman (2011) and Nomy Arpaly (2003) are advocates of such views. As Mason notes, their views differ in that Harman’s does not take epistemic error to be necessary for blameworthiness. One can be blameless as a believer, and still be blameworthy as a moral agent. Contrary to that, Arpaly has it that, in order to have a bad quality of will, an agent must have ignored evidence (2003: 104, Mason 2015: 3044). This seems to me to be on the right track. One concern with Harmann’s view is that it might not let *enough* people off the hook. Imagine that someone behaves epistemically in a way that fulfills reasonable expectations towards them: they consider evidence carefully and take other people’s testimonies seriously. In this case, it would be unfair to deem their ignorance culpable and thus to hold them responsible for ensuing actions.

This brings us to a third category of views, which take epistemic fault to be necessary for blameworthiness, where this epistemic fault need not be “freely and knowingly pass an opportunity for knowledge” (Levy 2011: 117) or the ignorance of relevant evidence. Michelle Moody-Adams (1994), Williams Fitzpatrick (2008, 2017) and Alex Guerrero (2007, 2017) have defended such views. I will focus on Fitzpatrick’s approach. On his view, the knowledge condition on moral responsibility should be spelled out as follows:

Ignorance, whether circumstantial or normative, is culpable if the agent could reasonably have been expected to take measures that would have corrected or avoided it, given his or her capabilities and the opportunities provided by the social context, but failed to do so either due to akrasia or due to the culpable, nonakratic exercices of such vices as overconfidence, arrogance, dismissiveness, laziness, dogmatism, incuriosity, self-indulgence, contempt and so on. (2008: 609).

He illustrates this with the case of a greedy businessman, Mr Potter, who engages in clearly morally objectionable practices in order to enhance profit and honestly thinks what he does is morally permissible. On the proposed view, Mr Potter is blameworthy because his ignorance is culpable to the extent that it stems from the above-mentioned vices. If he wasn’t, for instance, over-confident or dogmatic, he wouldn’t have believed that what he does is permissible. As Fitzpatrick notes, three aspects of Potter’s situation should lead us to conclude that his ignorance is culpable. First, there is “no relevant limitations in his social context” (2008: 605) that could have limited his abilities to reflect on these matters. Second, his failure to properly reflect on these issues is the result of “voluntary exercises of vices”. Finally, he could “reasonably have been expected to take steps that would have eliminated that ignorance, by refraining from exercising those vices and instead taking advantage of the epistemically relevant opportunities available to him” (2008: 605).

One might object that it is unreasonable to expect Mr. Potter to revise his belief, given that the people around him—such as his colleagues—share his view that their business practices are just “how business is done”. One could thus argue that Mr. Potter lacks epistemically relevant opportunities to reconsider his beliefs. However, this defense likely fails. If we assume that Mr. Potter consumes news media like many of us, he must be aware that businesses like his exploit vulnerable populations and contribute to the climate crisis. Additionally, it’s plausible that not all of Mr. Potter’s acquaintances share his views. Perhaps his stepbrother is an environmental activist, or his young cousin frequently posts about the human rights issues in fast fashion on Instagram. He may even witness protests in his town. Given these epistemic opportunities, Mr. Potter has more than enough access to information that could challenge his belief. Fitzpatrick’s view thus leads to the intuitive conclusion that Mr. Potter is blameworthy for his moral ignorance. One further advantage of this view is that its focus on reasonable demands is helpful in thinking about responsibility in the accountability sense as we have defined it: one is culpable for one’s ignorance in the accountability sense if it would be reasonable for others to expect him to e take measure to correct or avoid it.

Now, is Mario responsible for his moral ignorance when he fails to take responsibility for his actions? Again, the answer is likely to be no. Recall that the three criteria identified by Fitzpatrick for the culpable moral ignorance of Mr Potter are the following.

First, there is “no relevant limitations in his social context” (2008: 605) that could have limited his abilities to reflect on these matters. In Mario’s case, it is clear that *there are* such limitations in his social context. As I emphasized, it is important that individuals with BPD are diagnosed and receive proper therapeutic help to revise their problematic beliefs. As Mario is not diagnosed, there are limitations on his social context for understanding what is really going on.

The second criterion is that Mr. Potter’s failure to properly reflect on these issues is the result of the “voluntary exercises of vices”: if he didn’t have those vices, he would probably have different beliefs. By contrast with this, Mario’s moral ignorance does *not* stem from the voluntary exercise of vices such as arrogance or overconfidence. It is of course possible that there are arrogant people with BPD or that they sometimes are overconfident. However, even if they weren’t arrogant or overconfident, individuals with BPD are likely to maintain the same beliefs concerning the moral permissibility of their actions for the reasons we mentioned: because of their unstable sense of self (Leichsenring et al. 2024), they have a hard time recognizing their actions as morally wrong.One indication of this is found in a study by Peters et al. (2016), in which they found that individuals with BPD typically experience relatively low degrees of guilt compared to control groups. As I have explained, this is becauseadmitting that they did wrong, either to others or to themselves, would further threaten their internal coherence. There is thus a clear explanation as to why they regard their actions as incorrectly being permissible and harm their loved ones by not apologizing to them: their beliefs are not caused by the individual’s vices but by the well-known symptoms of their disorder.

Finally, the last criterion is that he could “reasonably have been expected to take steps that would have eliminated that ignorance, by refraining from exercising those vices and instead taking advantage of the epistemically relevant opportunities available to him” (2008: 605). Can we reasonably demand of Mario that he had different moral beliefs? Were there epistemically relevant opportunities available to him? In Mario’s specific case, the answer to the first question is “no” as it is said in the example that he does not receive appropriate therapeutic help.^9^ Further, it seems unreasonable to expect him to recognize the true moral nature of her actions when doing so is psychologically so costly for him.

## Conclusion

In this paper, I have explored the moral responsibility of individuals with BPD by focusing my discussion in two key aspects. First, I focused on a specific aspect of their behavior: their frequent expressions of anger and aggression. Second, rather than solely delving into the concept of control, I also examined the epistemic condition of moral responsibility. The overall conclusion is that BPD patients’ control and epistemic capacities are limited. In typical circumstances, we should thus generally renounce holding them morally responsible for their aggression to the same degree as we would hold responsible a neurotypical individual for their anger and aggressions. We should also keep in mind that capacities in regulating one’s belief and actions will vary from one individual to another, depending, for instance, on whether they receive appropriate treatment or not and on the gravity of the disorder. In ideal circumstance, when individuals with BPD receive proper care and information about their conditions from medical professional, they might become fitting targets for moderate attribution of moral responsibility.

## Footnotes

1 For instance, while manipulation is often associated with a borderline personality, it is not among its diagnosis criteria. For a discussion on BPD and manipulation, see Potter, Nancy Nyquist. ‘What Is Manipulative Behavior, Anyway?’ *Journal of Personality Disorders* 20, no. 2 (April 2006): 139–56; discussion 181-185. https://doi.org/10.1521/pedi.2006.20.2.139. Bowers (2003:323), quoted in Nyquist Potter (2006: 139) observes that “so frequently does the word crop up in conversations and discourses about such patients, that it might wrongly be thought of as the major problem that such patients pose to psychiatric services, or the major defining criteria of their disorders”.

2 As an example of this tendency, Sarah Sulzer, notes that “The very symptoms and behaviors associated with BPD are linked with how providers define the “difficult patient” (2015: 82).

3 American Psychiatric Association. (2013) borderline personality disorder. *Diagnostic and Statistical Manual of Mental Disorders*, 5th, ed. American Psychiatric Publishing. *DSM-V,* https://dsm-psychiatryonline-org.ruby.uhv.edu/doi/full/10.1176/appi.books.9780890425596.dsm05.

4 There might be social factors influencing the prevalence of BPD diagnosis in women, as well as important consequences to this asymmetry. For a recent discussion see Dorfman, Natalie, and Joel Michael Reynolds. ‘The New Hysteria: Borderline Personality Disorder and Epistemic Injustice’. *International Journal of Feminist Approaches to Bioethics* 16, no. 2 (2023): 162–81.

5 There are two main kinds of dichotomous thinking, unidimensional and multidimensional. Unidimensional dichotomous thinking is the tendency to make extreme evaluations where these evaluations are always either all good or all bad (Veen & Arntz 2000: 24). On the other hand, “multidimensional” dichotomous thinking is defined as the making of extreme evaluations with different valences on the positive–negative dimension. In this case, a person can be simultaneously evaluated as ‘‘totally reliable’’ and ‘‘totally lacking self-confidence’’ and is therefore not seen as ‘‘all good’’ or ‘‘all bad.’’ (Veen & Arntz 2000: 24). Extreme evaluations can explain that BPD patients react in extreme ways. (Sieswerda et al. 2013: 36).

6 See aksi Barnow et al., 2010

7 Quoted in Menges 2023 p. 616.

8 Although it is somehow difficult to find academic works on the topic, references to this difficulty abound on website dedicated to helping families cope with individuals with BPD. See for instance https://www.grouporttherapy.com/blog/borderline-personality-disorder-blaming-others. One can also find numerous discussions on Quora https://www.quora.com/Why-can-t-BPD-people-ever-admit-they-are-wrong and Reddit, of people living with individuals with BPD, testifying that their loved ones with BPD can never admit that they are wrong. It is surprising that not more academic work is devoted to this aspect of living with an individual with BPD. See also https://adrtimes.com/high-conflict-personality/ and https://www.psychologytoday.com/intl/blog/5-types-people-who-can-ruin-your-life/201803/are-you-target-blame-borderline-personality

9 Of course, depending on some circumstances such as his age, his education, his wealth, social context and so on, one could expect that Mario seeks therapeutic help by himself. It is thus difficult to render definitive verdict in the abstract.
